# Treatments for Relapsed-Refractory Diffuse Large B-cell Lymphoma: A Preliminary Evaluation of the Place in Therapy of Glofitamab, a Bispecific Monoclonal Antibody

**DOI:** 10.7759/cureus.33169

**Published:** 2022-12-31

**Authors:** Andrea Messori, Melania Rivano, Daniele Mengato, Marco Chiumente

**Affiliations:** 1 Health Technology Assessment (HTA) Unit, Regione Toscana, Firenze, ITA; 2 Clinical Oncology Pharmacy Department, Armando (A) Businco Hospital, Cagliari, ITA; 3 Hospital Pharmacy Department, Azienda Ospedale Università di Padova, Padova, ITA; 4 Scientific Direction, Italian Society for Clinical Pharmacy and Therapeutics, Milano, ITA

**Keywords:** selinexor, polatuzumab vedotin, loncastuximab tesirine, tafasitamab plus lenalidomide, glofitamab, overall survival, relapsed-refractory non-hodgkin lymphoma

## Abstract

Background and objectives

Glofitamab, tafasitamab, loncastuximab tesirine, polatuzumab, and selinexor have been proposed for the treatment of relapsed-refractory diffuse large B-cell lymphoma (DLBCL). We studied the pattern of overall survival (OS) for these five agents.

Methods

We reconstructed patient-level data from the Kaplan-Meier OS graphs published in five pivotal trials. For this purpose, we used an artificial intelligence technique (the Shiny method). Reconstructed survival curves were subjected to standard statistics to perform cross-trial indirect comparisons; medians and hazard ratios (HRs) with 95% confidence interval (CI) were estimated for each treatment.

Results

Using glofitamab (a bispecific antibody) as a common comparator, our analysis of OS yielded the following results: a) tafasitamab plus lenalidomide, HR: 0.514 (95% CI: 0.341 to 0.776; P=0.0015); b) polatuzumab vedotin, HR: 0.822 (95% CI: 0.509 to 1.327); c) selinexor, HR: 1.170 (95% CI: 0.852 to 1.603); and d) loncastuximab tesirine, HR: 1.120 (95% CI: 0.868 to 1.659). Medians were estimated as follows: a) tafasitamab plus lenalidomide, 26.5 months (95% CI: 18.9 to NA); b) polatuzumab vedotin, 12.5 months (95% CI: 9.03 to NA); c) glofitamab, 11.7 months (95% CI: 7.96 to 18.0); d) loncastuximab tesirine, 10.2 months (95% CI: 6.97 to 11.6); and e) selinexor, 10.1 months (95% CI: 6.72 to 14.2).

Conclusions

These comparative results represent an original finding generated by the Shiny method. Although these comparisons are indirect, our analysis offers a useful synthesis of the outcomes of these treatments. According to these results, glofitamab, despite its improved mechanism of action, does not seem to confer any OS advantage compared with the other four treatments.

## Introduction

More than 10 years ago, monoclonal antibodies revolutionized the treatment of numerous hematologic malignancies. More recently, the development of bispecific antibodies likely represents another breakthrough innovation in this field, even though the clinical evidence is still preliminary [1].

Glofitamab differs from other anti-cluster of differentiation 20 (CD20) and anti-cluster of differentiation 3 (CD3) antibodies in that it has two anti-CD20-binding domains; this agent is therefore bivalent for the CD20 tumor antigen and monovalent for the T-cell CD3 protein [1]. The clinical experience with glofitamab is currently limited to a phase 1 trial [2] and a phase 2 trial [3], both conducted in patients with relapsed or refractory diffuse large B-cell lymphoma (DLBCL). In the absence of head-to-head comparisons between glofitamab and standard agents, in the present study, we performed an indirect comparison between glofitamab and four standard agents for relapsed-refractory DLBCL. For this purpose, individual patient data from five trials were reconstructed using an artificial intelligence software (the Shiny method [4,5]) that has already been employed to evaluate the place in therapy of numerous innovative anticancer agents [6]. In July 2022, one of these cross-trial comparisons (denoted also as Shiny method reviews) has already been published on relapsed-refractory DLBCL [7]; thereafter, in December 2022, the pivotal phase 2 trial on glofitamab has been published [3], and this trial has further increased the interest of the scientific community for these innovative treatments aimed at this disease condition.

The pivotal trials that have studied these five agents are phase 2 and therefore did not include any control group. In the absence of controlled trials, indirect comparisons can provide useful information to evaluate these five agents comparatively and to determine their place in therapy. The Shiny method [4,5] is a new technique that performs indirect comparisons among treatments aimed at the same disease condition and that can be applied when the clinical end point is represented by overall survival (OS) or progression-free survival.

## Materials and methods

Study design

The present analysis was designed to study patients with relapsed-refractory DLBCL treated with five novel treatments (glofitamab, tafasitamab plus lenalidomide, loncastuximab tesirine, polatuzumab, or selinexor). The clinical material was represented by the pivotal trial published for each of these five agents; OS was the end point of the analysis. Our aim was to carry out some indirect cross-trial comparisons among these five treatments by the application of the Shiny method [4,5]. As previously pointed out, the analysis on tafasitamab plus lenalidomide, loncastuximab tesirine, polatuzumab, and selinexor has been published in a previous report [7].

Statistical analysis

For each clinical study, we analyzed the Kaplan-Meier graph of OS (along with the total number of enrolled patients and the total number of deaths). Then, for each OS curve, we reconstructed patient-level data from the graph using the Shiny method [4,5]. The graph of each Kaplan-Meier curve was digitized and converted into x-y data pairs using WebPlotDigitizer (Ankit Rohatgi, Pacifica, CA, USA). This combined application of WebPlotDigitizer and the Shiny software is well standardized [6]. Finally, the reconstructed survival curves for the five treatments were pooled into a single Kaplan-Meier graph, which was handled according to standard statistical analyses. Pairwise comparisons were handled by determining the hazard ratio (HR) along with 95% confidence interval (CI). Medians (with 95% CI) were also determined. Statistical significance was set at P<0.05. All calculations were performed using the R-platform [8]; three packages (“coxph,” “survfit,” and “ggsurvplot”) were used.

Patients​​​​’​ informed consent

Informed consent from patients was not necessary because our analysis relied on clinical material already published in previous reports.

## Results

**Figure 1:**
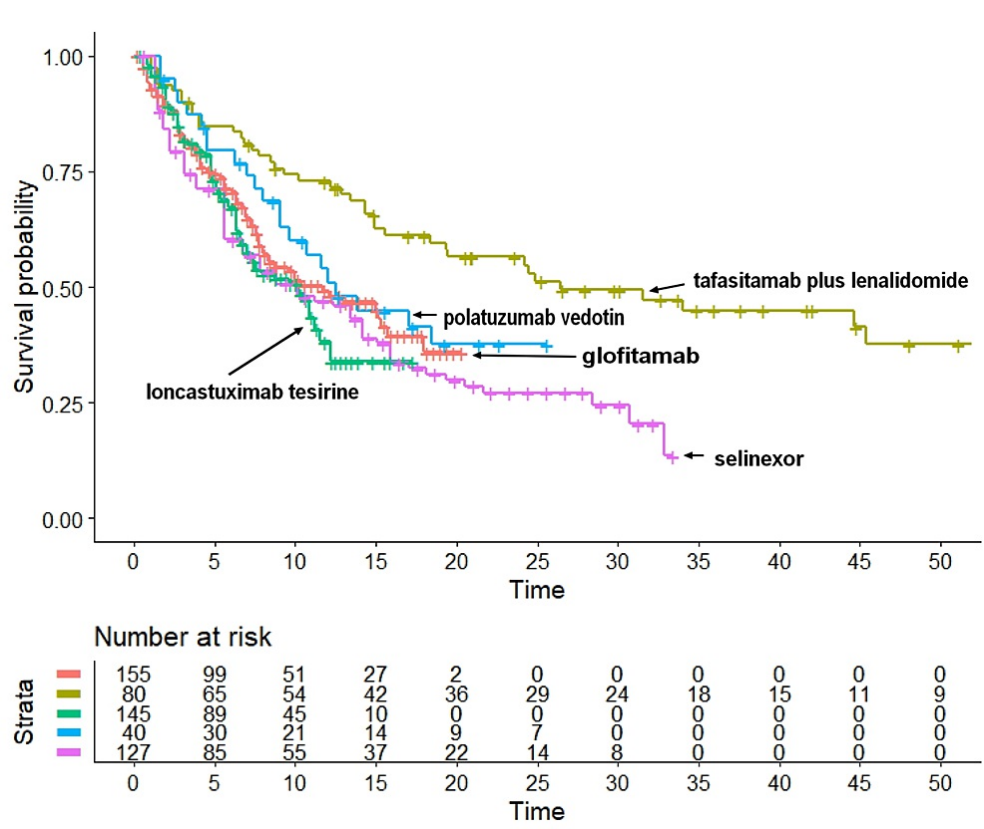
The five Kaplan-Meier curves refer to the following treatments: a) tafasitamab plus lenalidomide (in dark green), b) polatuzumab vedotin (in blue), c) glofitamab (in orange), d) loncastuximab tesirine (in green), and e) selinexor (in purple). End point and overall survival.

Table 1 illustrates the main characteristics of the five trials [3,9-12]. After reconstructing individual patient data from each trial according to the Shiny method, we generated the Kaplan-Meier curves of OS illustrated in Figure 1. The following median values of OS were estimated: a) tafasitamab plus lenalidomide, 26.5 months (95% CI: 18.39 to NA); b) polatuzumab vedotin, 12.5 months (95% CI: 9.03 to NA); c) glofitamab, 11.7 months (95% CI: 7.96 to 18.0); d) loncastuximab tesirine, 10.2 months (95% CI: 6.97 to 11.6); and e) selinexor, 10.1 months (95% CI: 6.72 to 14.2). According to these medians, tafasitamab plus lenalidomide ranked first in OS. The median OS for this treatment was significantly longer than that of polatuzumab vedotin, glofitamab, loncastuximab tesirine, and selinexor. In contrast, medians of OS did not differ significantly across polatuzumab vedotin, glofitamab, loncastuximab tesirine, and selinexor.

**Table 1:** Characteristics of the five phase 2 trials included in the analysis. ꝉPatients received pretreatment with obinutuzumab to mitigate cytokine release syndrome. §The inclusion criteria were revised while the study was ongoing (see the original paper for further details [9]). DLBCL, diffuse large B-cell lymphoma; CAR: chimeric antigen receptor

First author (year of publication)	Inclusion criteria	Treatment	Number of patients	Number of events	Percentage of patients treated with CAR T-cell therapy
Dickinson et al., 2022 [3]	Patients with relapsed or refractory DLBCL who had received at least two lines of therapy previously	Fixed-duration glofitamab monotherapy (12 cycles)ꝉ	155	58	33%
Duell et al., 2021 [9]	Patients with relapse or progressive disease and with Eastern Cooperative Oncology Group performance status of 0-2 who had received one to three prior systemic therapies§	Tafasitamab plus lenalidomide (up to 12 cycles)	80	48	0%
Sehn et al., 2020 [10]	Patients with transplantation-ineligible relapsed/refractory DLBCL who had received one to seven (median: two) prior systemic therapies	Polatuzumab vedotin	40	29	0%
Caimi et al., 2021 [11]	Patients aged 18 years or older with relapsed or refractory disease after two or more (median: three) multiagent systemic treatments who had measurable disease and Eastern Cooperative Oncology Group performance status of 0-2	Loncastuximab tesirine	145	77	9%
Kalakonda et al., 2020 [12]	Patients aged 18 years or older with pathologically confirmed DLBCL and an Eastern Cooperative Oncology Group performance status of 2 or less who had received two to five lines of previous therapies and progressed after or were not candidates for autologous stem-cell transplantation	Selinexor	127	73	0%

Regarding the HR for indirect pairwise comparisons between these agents, glofitamab was selected as a common comparator for the other four agents. Our pairwise comparisons yielded the following results: a) tafasitamab plus lenalidomide, HR: 0.514 (95% CI: 0.341 to 0.776); b) polatuzumab vedotin, HR: 0.822 (95% CI: 0.509 to 1.327); c) selinexor, HR: 1.169 (95% CI: 0.852 to 1.603); and d) loncastuximab tesirine, HR: 1.200 (95% CI: 0.8688 to 1.659). Hence, compared with glofitamab, tafasitamab plus lenalidomide showed a significantly better OS (P=0.0015), while polatuzumab vedotin fared numerically better than glofitamab but remained far from statistical significance (P=0.42). Finally, selinexor showed a worse OS than glofitamab, but the difference was not significant (P=0.33).

Indirect statistical comparisons are essential to appropriately interpret the descriptive results shown in Figure 1. As judged by the end point of OS, the ranking in medians was a very preliminary but useful information to compare the effectiveness of these five agents. Thereafter, the values of HR quantified these comparisons in more depth and provided the commonly used indexes of statistical significance based on the HR.

All in all, the basic results of our analysis were summarized by a few pieces of information: the Kaplan-Meier graphs of OS and the values of HR and medians. Interestingly enough, while bispecific antibodies were expected to determine a better OS than the four comparators, no improvement in survival was observed. Rather, tafasitamab plus lenalidomide was found to determine a highly significantly better OS than glofitamab.

To explain this unexpected finding, we extracted from the five trials one important prognostic characteristic at baseline represented by the previous therapy with a chimeric antigen receptor (CAR) T-cell product, which can be assumed to be an unfavorable prognostic factor. At the bottom of Table 1, the percentage of patients previously treated with a CAR T-cell product is reported for each of the five trials; this percentage spanned from 0% for tafasitamab plus lenalidomide, polatuzumab vedotin, and selinexor to 9% for loncastuximab tesirine, to 33% for glofitamab. These differences support the hypothesis that the patients enrolled by Dickinson et al. and treated with glofitamab [3] had a worse prognosis than the patients enrolled in the other four trials.

## Discussion

The main original finding of this study is represented by the cross-trial Kaplan-Meier graph (Figure 1), in which the OS pattern has been reported for each of the five treatments. In the first place, the visual inspection of this graph permits to rank the effectiveness across the five treatments and, more importantly, to evaluate the clinical relevance of survival differences along with their statistical significance.

Our results clearly favor the combination of tafasitamab and lenalidomide. Although this finding must be viewed with caution owing to the indirect nature of the comparisons, the magnitude of incremental benefit for tafasitamab plus lenalidomide was remarkable, which explains the rationale for undertaking future trials of direct comparison based on this combination treatment. On the other hand, one should keep in mind that the patients of Duell et al.’s trial, treated with tafasitamab, had received only one to three previous lines of systemic treatment, whereas some of the four comparators had been preceded by numerous previous lines (up to seven for Sehn et al.’s trial and up to five for Kalakonda et al.’s trial).

The differences in OS that we found might depend on intrinsic differences in effectiveness across the treatments or on different characteristics of the enrolled patients. It is most likely that both factors played a role. Regarding the patient eligibility criteria, these were very similar in the five trials but not the same; therefore, the patient cohorts included in our analysis likely did not have the same risk or prognosis, as suggested by the different number of previous lines of treatment. However, the extent to which this factor contributed to the final results remains unknown.

One important advantage of the Shiny method [4,5] lies in its ability to evaluate the time course of the survival pattern, which is a fundamental piece of information. In contrast, in a standard meta-analysis, where the survival differences are expressed through a forest plot, no information is generated about the survival trends over time. While, in both cases, the HRs provide the final results, the advantage of the Shiny method is that the Kaplan-Meier graphs convey much more information than the forest plot.

The present work has the typical limitations of all analyses that are based on the Shiny method [6,13-15]. Among these, the most relevant is that the comparisons are indirect, and so, the results might be affected by differences in the characteristics of the original patient cohorts. Regarding the percentage of patients previously treated with a CAR T-cell product, the worse outcome found with glofitamab compared with tafasitamab plus lenalidomide has likely been influenced by the worse prognosis of the patients enrolled by Dickinson et al., as witnessed by the frequent use of CAR T-cells.

All in all, interpreting these findings is not straightforward because one hypothesis is that the “true” efficacy of glofitamab is less than expected. Another explanation is that the patients studied by Dickinson et al. had intrinsically worse prognosis compared with the patients enrolled in the other four studies. Further data are needed to share light on this question.

## Conclusions

The experience presented herein confirms the feasibility of reconstructing patient-level data from Kaplan-Meier graphs in order to generate survival statistics and synthesize clinical evidence. The results of our statistical comparisons represent an original finding that summarizes the evidence currently available for these five innovative treatments in relapsed-refractory DLBCL. In this disease condition, CAR T-cells are known to offer an important alternative to the five treatments examined in this article. An analysis based on the Shiny method has recently been published regarding CAR T-cell products in relapsed-refractory DLBCL. The present article focused on pharmacological treatments offers a complete and updated therapeutic landscape about relapsed-refractory DLBCL.

## References

[1] Longo DL (2022). The expanding clinical role of bifunctional antibodies. N Engl J Med.

[2] Hutchings M, Morschhauser F, Iacoboni G (2021). Glofitamab, a novel, bivalent CD20-targeting T-cell-engaging bispecific antibody, induces durable complete remissions in relapsed or refractory B-cell lymphoma: a phase I trial. J Clin Oncol.

[3] Dickinson MJ, Carlo-Stella C, Morschhauser F (2022). Glofitamab for relapsed or refractory diffuse large B-cell lymphoma. N Engl J Med.

[4] Liu N, Zhou Y, Lee JJ (2021). IPDfromKM: reconstruct individual patient data from published Kaplan-Meier survival curves. BMC Med Res Methodol.

[5] Messori A (2021). Synthetizing published evidence on survival by reconstruction of patient-level data and generation of a multi-trial Kaplan-Meier curve. Cureus.

[6] Messori A (2022). OSFHOME: application of the shiny method in the analysis of survival curves: a synopsis of 18 references. Open Science Framework, 2022, published 15 August.

[7] Messori A, Caccese E (2022). Treatments for relapsed-refractory diffuse large B-cell lymphoma: comparison of overall survival outcomes observed with four novel agents. Eur Rev Med Pharmacol Sci.

[8] R Core Team (2013). R: a language and environment for statistical computing.

[9] Duell J, Maddocks KJ, González-Barca E (2021). Long-term outcomes from the phase II L-MIND study of tafasitamab (MOR208) plus lenalidomide in patients with relapsed or refractory diffuse large B-cell lymphoma. Haematologica.

[10] Sehn LH, Herrera AF, Flowers CR (2020). Polatuzumab vedotin in relapsed or refractory diffuse large B-cell lymphoma. J Clin Oncol.

[11] Caimi PF, Ai W, Alderuccio JP (2021). Loncastuximab tesirine in relapsed or refractory diffuse large B-cell lymphoma (LOTIS- 2): a multicentre, open-label, single-arm, phase 2 trial. Lancet Oncol.

[12] Kalakonda N, Maerevoet M, Cavallo F (2020). Selinexor in patients with relapsed or refractory diffuse large B-cell lymphoma (SADAL): a single-arm, multinational, multicentre, open-label, phase 2 trial. Lancet Haematol.

[13] Messori A, Chiumente M, Mengato D (2022). Chimeric antigen receptor T cells in large B-cell lymphoma: analysis of overall survival based on reconstructed patient-level data. Clin Ther.

[14] Messori A (2023). Long-term progression-free survival in patients with chronic lymphocytic leukemia treated with novel agents: an analysis based on indirect comparisons. Eur J Haematol.

[15] Ossato A, Damuzzo V, Baldo P, Mengato D, Chiumente M, Messori A (2022). Immune checkpoint inhibitors as first line in advanced melanoma: evaluating progression-free survival based on reconstructed individual patient data. Cancer Med.

